# *Lacticaseibacillus rhamnosus* Hao9 exerts antidiabetic effects by regulating gut microbiome, glucagon metabolism, and insulin levels in type 2 diabetic mice

**DOI:** 10.3389/fnut.2022.1081778

**Published:** 2023-01-05

**Authors:** Mei Han, Wenyan Liao, Yao Dong, Chen Bai, Zhonghui Gai

**Affiliations:** ^1^Department of Food Science, Shanghai Business School, Shanghai, China; ^2^State Key Laboratory of Dairy Biotechnology, Shanghai Engineering Center of Dairy Biotechnology, Bright Dairy & Food Co., Ltd., Shanghai, China; ^3^Department of Research and Development, Wecare Probiotics (Suzhou) Co., Ltd., Suzhou, China

**Keywords:** type 2 diabetes mellitus, *Lacticaseibacillus rhamnosus* Hao9, gut microbiota, liver ROS, serum inflammatory factors

## Abstract

**Introduction:**

Type 2 diabetes mellitus (T2DM) is a metabolic disease that has led to a significant global public health burden.

**Methods:**

In this work, we investigated the effects of *Lacticaseibacillus rhamnosus* Hao9 on T2DM in mice with high-fat diet- and streptozotocin (STZ)-induced diabetes (diabetic mice) and explored the underlying mechanisms.

**Results:**

We found that 10^9^ colony forming units (CFUs) of Hao9 per day significantly reduced fasting blood glucose and insulin levels (*p* < 0.001) in diabetic mice. Moreover, Hao9 enhanced liver antioxidant capacity and significantly decreased glucose-6-phosphatase and phosphoenolpyruvate carboxykinase expression in the livers of diabetic mice (*p* < 0.001). Hao9 also reduced the serum concentrations of pro-inflammatory cytokines such as tumor necrosis factor alpha (TNFα), interleukin-1β (IL1β), and IL6 (*p* < 0.05) and improved intestinal barrier function in diabetic mice. The composition of the gut microbiome was modulated by Hao9, with an increased abundance of *Roseburia, Eubacterium*, and *Lacticaseibacillus*, and decreased abundance of *Escherichia/Shigella*. Notably, *Lacticaseibacillus* was detected at both weeks 5 and 12 post-treatment, suggesting sustained colonization of the gut by Hao9.

**Discussion:**

The supplementation of Hao9 improved gut microbiota, glucose metabolism, and insulin levels significantly in T2DM mice. That means Hao9 contributes to improving T2DM symptoms with its potential beneficial effects. Therefore, Hao9 is a promising dietary supplement for the treatment of T2DM.

## Introduction

Type 2 diabetes mellitus (T2DM) is a chronic metabolic disease that has led to a significant global public health burden (1). Several studies have shown that dysbiosis of the gut microbiome can influence inflammation, insulin resistance, and incretin secretion (2–5). The gut microbiome of T2DM patients is characterized by reduced numbers of short-chain fatty acid (SCFA)-producing bacteria and an enrichment in opportunistic pathogens. Moreover, dysregulation of the gut microbiome has been associated with both the development and progression of T2DM (2, 6), making the gut microbiota a promising novel target for the management of T2DM (6).

Recently, studies have investigated the beneficial effects of probiotics on the gut microbiome. *Bifidobacterium* and *Lactobacillus* are commonly used probiotics with anti-inflammatory, hypolipidemic, hypoglycemic, and immunomodulatory properties (7, 8). Both clinical and animal studies have shown that appropriate intake of probiotics can alleviate a variety of chronic diseases, including T2DM. For example, studies have shown that *Bifidobacterium longum* subsp. *longum* BL21 (9), *Lactobacillus casei* CCFM419 (10), and *Lactobacillus casei* LC89 (11) can reduce insulin resistance and hyperglycemia. Clinical studies have shown that *Lactobacillus acidophilus* La5 and *Bifidobacterium animalis* subsp. *lactis* Bb12 can improve insulin sensitivity (12). Randomized clinical trials have also shown modest improvements in insulin resistance in patients with T2DM receiving mixture probiotics of *Bifidobacterium*, *Lactobacillus, Lactococcus*, and *Propionibacterium* (13). Although the antidiabetic effects of probiotics are clear, they can have strain-specific effects and may use different mechanisms to achieve the same effects (14). Therefore, it is necessary to identify novel probiotic strains with functional specificity for the treatment of T2DM.

In our previous study, *Lacticaseibacillus rhamnosus* Hao9 was found to positively affect both the gut microbiome and inflammatory abnormalities in mice with dextran sulfate sodium (DSS)-induced colitis (15). In addition, we demonstrated the safety and beneficial effects of fermented milk containing Hao9 on the gut microbiome and neurotransmitters in healthy mice. In view of this modulatory effect of Hao9 on the gut microbiome, the aim of this study was to investigate the effects of Hao9 in mice with high-fat diet (HFD)- and streptozotocin (STZ)-induced T2DM and identify any potential underlying mechanisms. We provide important insights into Hao9-medicated improvements in T2DM through regulation of gluconeogenesis-specific gene expression and the gut microbiome composition.

## Materials and methods

### Preparation of *Lacticaseibacillus rhamnosus* Hao9 bacterial suspension

*Lacticaseibacillus rhamnosus* Hao9 was cultured in De Man, Rogosa and Sharp (MRS) broth for 24 h at 37°C. Bacterial cells were collected by centrifugation (4,500 × *g*, 10 min, 4°C) and the cell pellet was resuspended in sterile water to a final concentration of 5 × 10^9^ CFU/mL. Bacterial suspensions were prepared daily throughout the experiment.

### Animals and experimental design

Male C57BL/6J (6-week-old) specific-pathogen-free (SPF) mice were provided by Shanghai Laboratory Animal Center. Mice were maintained at a temperature of 22 ± 2°C, 55 ± 5% humidity, and a 12-h light/dark cycle, with food and water freely available throughout the experiment. All procedures involving mice conformed to the guidelines provided by Shanghai Laboratory Animal Center for Animal Care and Animal Experimentation under license number 2022032002.

**FIGURE 1:**
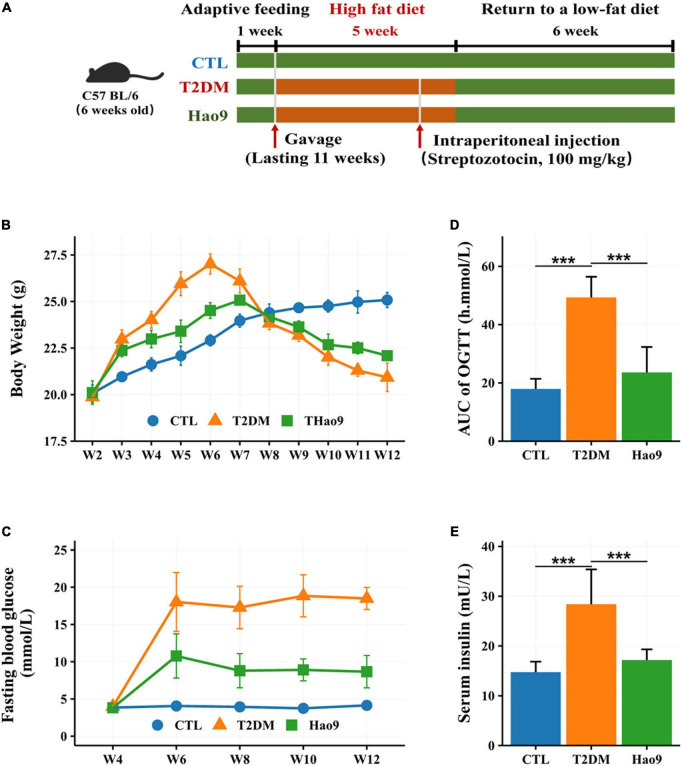
The experimental design of mice exposure to high fat diet (HFD) and streptozotocin (STZ) and treated with *L. rhamnosus* Hao9 **(A)**. The effect of Hao9 on body weight **(B)**, fasting blood glucose **(C)**, oral glucose tolerance **(D)**, and serum insulin levels **(E)** in HFD + STZ-induced diabetic mice. In bar plots **(D,E)** we used mean ± standard deviation (SD) to describe differences between groups and ***p < 0.001.

The experimental design is shown in Figure 1A. Twenty-four mice weighing 19–21 g were adaptively fed for 1 week and then divided into three groups of 8 mice each. The control group (CTL group) was fed a standard diet (350 Kcal/100 g) throughout the study and the T2DM and Hao9 groups were fed a HFD (455 Kcal/100 g) for 5 weeks before returning to a normal low-fat diet (16). In the second week, intragastric administration was started, in which 0.2 mL sterile water was administered alone in the T2DM and CTL groups, and 0.2 mL (5 × 10^9^ CFU/mL) Hao9 intragastric administration was used in the Hao9 group. In the fifth week, mice in the T2DM and Hao9 groups were injected intraperitoneally with STZ (Sigma-Aldrich) dissolved in buffer at a dose of 100 mg/kg, and CTL group was injected intraperitoneally with citrate buffer only. Five days after STZ injection, fasting blood glucose (FBG) was measured in T2DM and Hao9 groups, and mice with FBG levels higher than 7.1 mmol/L were considered T2DM. During the experiment, mice were weighed weekly and blood samples were collected from the tail vein every 2 weeks. FBG was measured using Accu-Chek Performa test strips (Roche).

### Oral glucose tolerance test

An oral glucose tolerance test (OGTT) was carried out at the end of the experiment, as previously described (17). Briefly, mice were fasted for 12 h, and blood glucose levels were measured using a glucometer at 30, 60, and 120 min after oral administration of a 2 g/kg glucose solution. The area under the glucose curve (AUC) was calculated.

### Collection of blood, liver and colon tissue, and feces

At the end of the experiment, mice were anesthetized with sodium pentobarbital (200 mg/kg i.p.) and sacrificed by cervical dislocation. Orbital blood samples were collected and centrifuged to obtain serum (4,000 × *g*, 10 min). Serum samples were then stored at −80°C. Liver samples were excised immediately and stored at −80°C. Colon specimens were fixed in 4% formaldehyde solution until histological analysis. Feces were collected in sterile tubes and stored at −80°C.

### Histopathological examination

Colon and liver tissues were fixed, dehydrated, and embedded in paraffin. Sections of 5-μm thickness were cut and stained using Masson’s trichrome stain. To assess hepatic reactive oxygen species (ROS) levels, frozen liver sections (4 μm) were stained with dihydroethidium (DHE). Paraffin-embedded colon tissue was cut into 5-mm sections and stained with periodic acid-Schiff (PAS) to visualize goblet cells.

### Biochemical analysis

One gram of liver tissue was collected from each group of mice and homogenized in 9 mL of normal saline in an ice bath. Liver superoxide dismutase (SOD) and catalase (CAT) activities were quantified using an enzyme-linked immunosorbent assay (ELISA) kit (Wuhan Chundu Biotechnology Co., Ltd., China).

Serum insulin, lipopolysaccharide (LPS), tumor necrosis factor alpha (TNFα), interleukin-1β (IL1β), IL6, and IL10 concentrations were measured using ELISA kits (Wuhan Chundu Biotechnology Co., Ltd., China). All assays were performed according to the manufacturer’s instructions.

### Colonic tight junction protein expression analysis

One gram of colon from each group of mice was collected and homogenized in 9 mL of normal saline in an ice bath to prepare a 10% colon tissue homogenate. Occludin, zonula occludens-1 (ZO-1), and claudin protein concentrations in the colonic homogenate were determined using ELISA kits (Wuhan Chundu Biotechnology Co., Ltd., China) per the manufacturer’s instructions.

### RNA isolation and RT-PCR analysis

Total RNA was isolated from 100 mg liver tissue using TRIzol reagent (Invitrogen, Thermo Fisher Scientific, USA), diluted to 1000 ng and used to synthesize cDNA using a reverse transcription polymerase chain reaction (RT-PCR) kit. Phosphoenolpyruvate carboxykinase (PEPCK) was amplified using forward primer 5′-CTGCATAACGGTCTGGACTTC-3′ and reverse primer 5′-CAGCAACTGCCCGTACTCC-3′, and glucose 6-phosphatase (G6Pase) was amplified using forward primer 5′-CGACTCGCTATCTCCAAGTGA-3′ and reverse primer 5′-GTTGAACCAGTCTCCGACCA-3′. Real-time PCR analysis was performed using an ABI 7900HT (Applied Biosystems, USA) platform and 100 ng cDNA. Gene expression levels were analyzed using the 2^–^^ΔΔ^ ^Ct^ method.

### DNA extraction and 16S rRNA gene sequencing analysis

Extraction of fecal DNA and amplification of the V3–V4 region of 16S rRNA was performed as previously reported (15, 18). Purified PCR products were sequenced using the Illumina MiSeq PE300 platform (Illumina, USA). Sequencing data were analyzed using Usearch (Version 11.0.667),^1^ and amplicon sequence variants (ASVs) tables were generated using USEARCH -unoise3 method. Representative sequences of ASVs were aligned to the 16S database using the RDP classifier (Version 18),^2^ for taxonomic classification. Alpha diversity was analyzed using vegan package (19).

### Statistical analysis

Gut microbiota with significant differences in abundance between experimental samples were identified using the non-parametric Kruskal–Wallis sum-rank test. Other continuous data were analyzed using one-way analysis of variance (ANOVA), and significant differences between groups were assessed by Bonferroni *post-hoc* test. Beta diversity between groups was assessed using principal component analysis (PCoA) and tested for significance using the adonis2 function of the R package vegan (19). Linear discriminant analysis effect size (LEfSe) analysis was performed to identify taxa with the greatest differences in abundance between the groups (20). Correlation analysis of the gut microbiome composition based on Bray–Curtis distance was performed using the Mantel test (19). All figures were generated using ggplot2 in R (21). In this study, we used median (interquartile range) to describe the relative abundance of gut microbiota at the genus level and mean ± standard deviation (SD) to describe other data such as blood indicators. Statistical analysis was performed using R4.2 (22), and *p*-values < 0.05 were considered statistically significant.

## Results

### Effect of Hao9 on body weight

Prior to STZ administration, mice in all groups gained weight on the HFD. However, the T2DM and Hao9 groups gained significantly more weight than the CTL group (Figure 1B). By week four, mice in the Hao9 group had significantly lower body weights than those in the T2DM group. Subsequently, exposure to STZ resulted in considerable weight loss in the T2DM group, whereas mice in the Hao9 group gained weight relative to those in the T2DM group. This suggests that administration of Hao9 broadly relieves this symptom of T2DM in mice.

### Effects of Hao9 on FBG, OGTT, and insulin levels

FBG levels were significantly higher in the T2DM group than in the CTL group, while administration of Hao9 significantly decreased FBG levels (Figure 1C). To determine the effect of Hao9 on glucose tolerance, an OGTT was performed at the end of the experiment (week 12). Hao9 mice had significantly decreased glucose AUC values over the course of the test, compared to T2DM mice (Figure 1D, *p* < 0.001). In addition, the Hao9 group had significantly decreased serum insulin levels compared with the T2DM group (Figure 1E, *p* < 0.001). These results suggest that Hao9 exerts hypoglycemic effects and can ameliorate glucose intolerance in HFD + STZ-induced T2DM mice.

### Protective effects of Hao9 on liver ROS and hepatic gluconeogenesis

**FIGURE 2:**
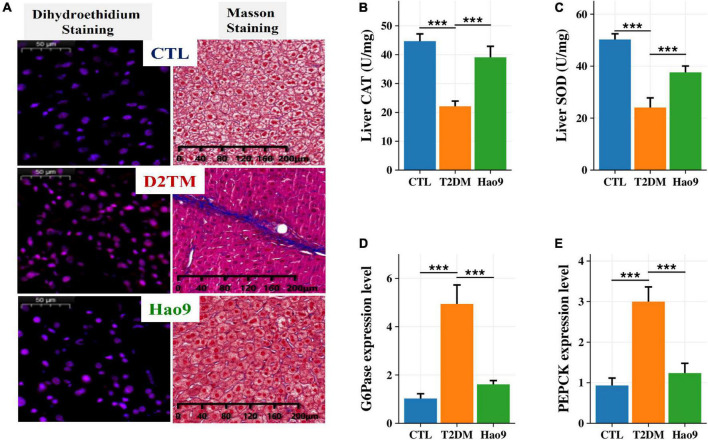
Protective effect of Hao9 on liver and hepatic glucose metabolism in T2DM mice. **(A)** Dihydroethidium (DHE) staining showed that Hao9 ameliorated hepatic reactive oxygen species, and Masson staining showed that Hao9 ameliorated the increase of collagen fibers in liver tissue caused by T2DM. Effect of Hao9 on hepatic catalase (CAT) **(B)** and superoxide dismutase (SOD) **(C)** levels. Effect of Hao9 on glucose-6-phosphatase (G6Pase) **(D)** and phosphoenolpyruvate carboxykinase (PEPCK) **(E)** mRNA expression in liver. In bar plots **(B–E)** we used mean ± standard deviation (SD) to describe differences between groups and ***p < 0.001.

DHE staining of liver tissue revealed increased ROS levels in the livers from the T2DM group relative to both the Hao9 and CTL groups (Figure 2A). In addition, Masson’s trichrome staining revealed that Hao9 supplementation increased glycogen storage in liver tissue and reduced liver fibrosis. These results suggest that Hao9 could reduce liver injury in T2DM mice.

Additionally, the hepatic CAT and SOD concentrations were significantly lower in T2DM mice than in CTL mice. Hao9 significantly rescued these hepatic CAT and SOD levels in T2DM mice (Figures 2B, C, *p* < 0.001).

To investigate the effect of Hao9 on hepatic gluconeogenesis, the hepatic expression of glucose metabolism-related genes was examined. Compared with the CTL group, the hepatic levels of mRNAs encoding G6Pase and PEPCK were increased in T2DM mice (Figures 2D, E, *p* < 0.001). Hao9 significantly reduced the expression levels of these genes, suggesting that Hao9 can regulate gluconeogenesis and exert an anti-hyperglycemic effect in T2DM mice.

### Effect of Hao9 on serum inflammatory factors and intestinal barrier integrity

**FIGURE 3:**
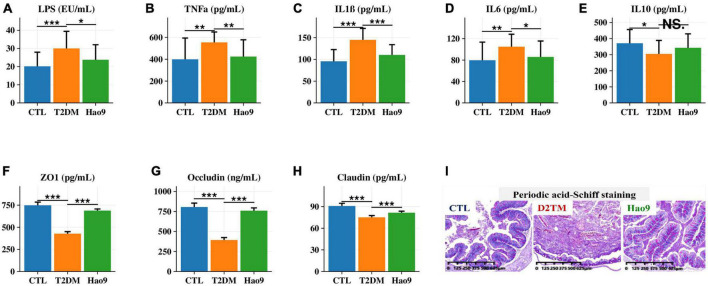
Effect of Hao9 on serum inflammatory factors and intestinal barrier. Effect of Hao9 on serum lipopolysaccharide (LPS) **(A)**, tumor necrosis factor alpha (TNFα) **(B)**, interleukin-1β (IL1β) **(C)**, IL6 **(D)**, and IL10 **(E)**. Effect of Hao9 on colonic zonula occludens (ZO-1) **(F)**, occludin **(G)**, and claudin **(H)**. Periodic acid-Schiff staining **(I)** showed the effect of Hao9 on histopathological changes in the colon of type 2 diabetes mellitus (T2DM) mice. *p < 0.05, **p < 0.01 and ***p < 0.001 vs. T2DM group. In bar plots, we used mean ± standard deviation (SD) to describe differences between groups.

Serum LPS, TNFα, IL1β, and IL6 concentrations were increased in the T2DM group compared to the CTL group. In contrast, Hao9 supplementation significantly reduced these pro-inflammatory factor concentrations (Figures 3A–D, *p* < 0.05). In addition, T2DM resulted in a significant decrease in serum IL10 levels, with Hao9 supplementation resulting in a positive but not significant increase (Figure 3E, *p* = 0.076).

Because compromised intestinal barriers often lead to the entry of pro-inflammatory factors, such as LPS, into the liver, we analyzed the effect of Hao9 on intestinal barrier integrity. Compared to the CTL group, the protein concentrations of occludin, ZO-1 and claudin were significantly decreased in the colons of mice in the T2DM group, while Hao9 supplementation significantly increased the expression of these essential tight junction proteins (Figures 3F-H). PAS staining of the colonic tissue revealed an intact crypt structure and abundant goblet cells in CTL mice (Figure 3I). In contrast, T2DM mice had disturbed colonic tissue integrity and depleted goblet cells. Compared with CTL group (65 ± 8/μm^2^), the number of goblet cells in T2DM mice (45 ± 6/μm^2^) was significantly reduced, but after the intervention of Hao9, the goblet cells number (72 ± 9/μm^2^) increased significantly (*p* = 0.015). The results suggested that Hao9 treatment increased the number of goblet cells and mucus secretion and reduced inflammatory cell infiltration into the intestinal mucosa (Supplementary Figure 1).

### Effects of Hao9 on gut microbiome composition

**FIGURE 4:**
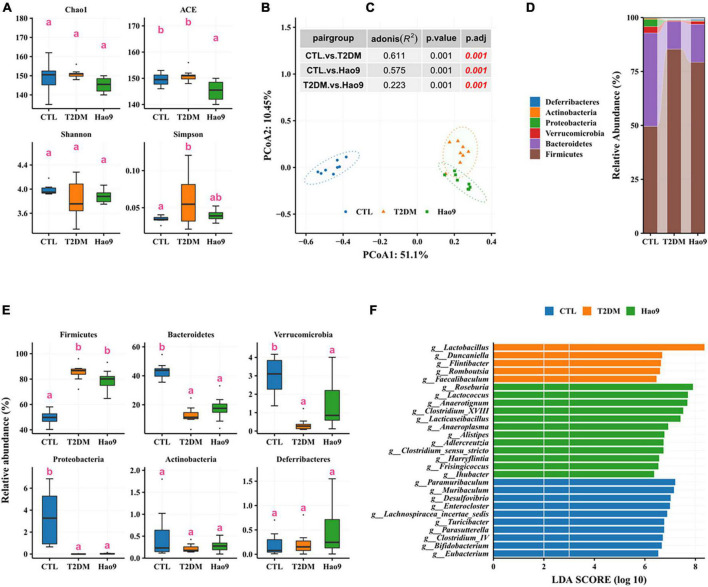
Effect of Hao9 on gut microbiota in type 2 diabetes mellitus (T2DM) mice after streptozotocin injection (week 5). Alpha diversity of gut microbiota in mice **(A)**. Principal coordinate analysis (PCoA) of gut microbiota based on Bray–Curtis distances **(B)**; table **(C)** in the figure shows the results of the adonis analysis between pair groups. Relative abundance of gut microbiota at phylum level **(D,E)**. Linear discriminant analysis effect size (LEfSe) was used to analyze the relative abundance **(F)** of gut microbiota at the genus level. Different superscript letters were statistically significantly different (*p* < 0.05).

To determine the effect of *L. rhamnosus* Hao9 on the gut microbiome composition, the bacterial compositions of fecal samples collected on weeks 5 and 12 were analyzed. At week 5, as reflected in the ACE index, there was no significant difference in microbial richness in the T2DM group compared to the CTL group, whereas it decreased in the Hao9 group (Figure 4A). In addition, gut microbial diversity was decreased in the T2DM group compared to the CTL group, as indicated by an increase of the Simpson index. PCoA analysis based on Bray–Curtis dissimilarity revealed a clear separation between the three groups (Figure 4B, C), suggesting that Hao9 remodels the structure of the gut microbiome in T2DM mice.

In addition, the gut microbiomes of mice in all groups consisted predominantly of Firmicutes and Bacteroidetes (Figure 4D). T2DM resulted in an increase in Firmicutes and a decrease in Bacteroidetes, Verrucomicrobia, and Proteobacteria, whereas Hao9 intervention tended to ameliorate these changes in a non-significant manner (Figure 4E). Mice receiving the Hao9 probiotic displayed no significant differences in Actinobacteria and Deferribacteres.

LEfSe analysis showed enrichment of *Lactobacillus*, *Duncaniella*, *Flintibacter*, *Romboutsia*, and *Faecalibaculum* in the T2DM group. However, Hao9 significantly increased the abundance of *Roseburia*, *Lactococcus*, *Anaerotignum*, *Clostridium*_XVIII, and *Lacticaseibacillus* in T2DM mice (Figure 4F and Supplementary Table 1). In addition, enrichment of *Eubacterium*, *Bifidobacterium*, and *Clostridium_*IV was seen in the CTL group.

**FIGURE 5:**
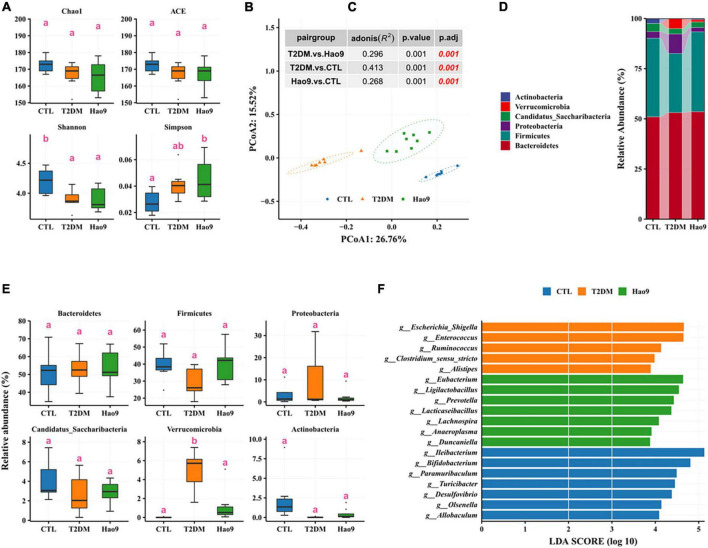
Effect of Hao9 on gut microbiota in type 2 diabetes mellitus (T2DM) mice at the end of the experiment (week 12). Alpha diversity of gut microbiota in T2DM mice **(A)**. Principal coordinate analysis (PCoA) based on distance **(B)**; the table **(C)** in the figure showed the results of the adonis significance analysis between groups. Relative abundance of gut microbiota at phylum level **(D,E)**. Linear discriminant analysis effect size (LEfSe) analysis between pair groups was used to analyze the relative abundance **(F)** of gut microbiota at the genus level. Different superscript letters denote groups that are statistically different (*p* < 0.05).

By week 12 no significant changes in microbial richness could be seen in the T2DM and Hao9 groups compared to the CTL group (Figure 5A). However, microbial diversity was decreased in both T2DM and Hao9 groups compared to the CTL group. As at week 5, PCoA analysis based on Bray–Curtis dissimilarity indicated a clear separation between the three groups (Figures 5B, C), indicating that Hao9 had a sustained and significant effect on the structure of the gut microbiome in T2DM mice.

Similar to the composition of the gut microbiota at week 5, the gut microbiota of mice at week 12 across all groups consisted predominantly of Firmicutes and Bacteroidetes (Figure 5D). The abundance of Verrucomicrobia was significantly increased in the T2DM group, whereas other phyla were not significantly altered (Figure 5E). In addition, Firmicutes decreased and Proteobacteria increased in the T2DM group.

LEfSe analysis showed enrichment of *Escherichia/Shigella*, *Enterococcus*, *Ruminococcus*, *Clostridium_sensu_stricto*, and *Alistipes* in the T2DM group. However, Hao9 significantly increased the relative abundance of *Eubacterium*, *Ligilactobacillus*, *Prevotella*, *Lacticaseibacillus*, and *Lachnospira* (Figure 5F and Supplementary Table 2). In addition, enrichment of *Ileibacterium*, *Bifidobacterium*, and *Paramuribaculum* was seen in the CTL group.

**FIGURE 6:**
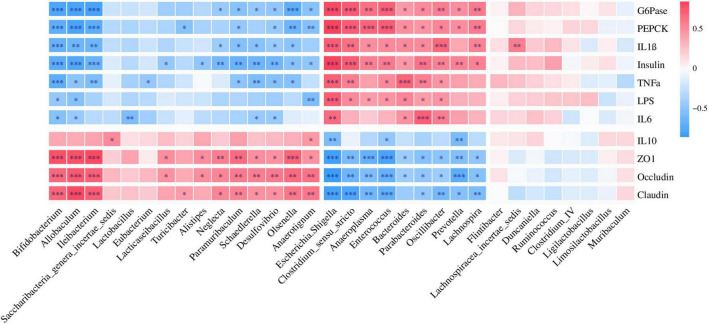
Correlations analysis between the genus level of gut microbiota and parameters related to glucose metabolism, cytokines, and colonic tight junction protein.

Correlation analysis revealed that *Ileibacterium*, *Bifidobacterium*, and *Paramuribaculum* enriched in the CTL group were positively correlated with ZO1, occludin, and claudin, but negatively correlated with Insulin, G6Pase, and PEPCK (Figure 6), which indicated that T2DM resulted in a decrease in the relative abundance of beneficial bacteria in the intestine. *Escherichia/Shigella* and *Enterococcus* enriched in T2DM were positively correlated with Insulin, G6Pase, and PEPCK, but negatively correlated with ZO1, Occludin, and Claudin (Figure 6). In addition, *Prevotella* and *Lachnospira* enriched in the Hao9 group were also positively correlated with Insulin, G6Pase and PEPCK, but negatively correlated with ZO1, occludin and claudin (Figure 6), which indicated that Hao9 could only partially restore gut microbiota disturbances caused by T2DM. Finally, we observed that *Lacticaseibacillus* enriched in the Hao9 group was positively correlated with ZO1, occludin, and negatively correlated with Insulin.

### Temporal correlation of mouse gut microbiota during probiotic treatment

**FIGURE 7:**
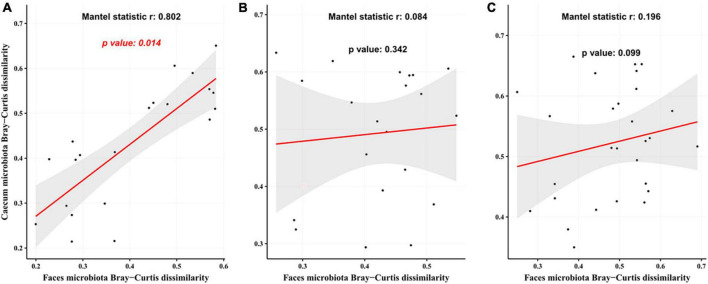
Correlation analysis of gut microbiota at the end of modeling (week 5) and at the end of the experiment (week 12) based on Bray–Curtis distance matrix. The analysis used the mantel function in vegan package using 999 permutations. **(A)** The control group (CTL) group; **(B)** type 2 diabetes mellitus (T2DM) group; **(C)** Hao9 group.

The Mantel test revealed a significant correlation between gut microbiota in mice at weeks 5 and 12 (Figure 7A, *r* = 0.802, *p* = 0.014) in the CTL group. This association was not seen in the T2DM or Hao9 groups (*p* > 0.05) (Figures 7B, C). However, a larger *r* value and a smaller *p*-value was calculated for the Hao9 group than for the T2DM group, suggesting rectification of the intestinal microbiome by Hao9.

## Discussion

Dietary interventions are a commonly used strategy for treating T2DM by modulating the structure of the gut microbiome. We investigated the potential of *L. rhamnosus* Hao9 to ameliorate the symptoms and gut dysbiosis seen in T2DM using mice with HFD + STZ-induced T2DM. We then explored the potential mechanisms by which Hao9 could exert beneficial effects on T2DM. Hao9 significantly increased body weight and decreased FBG levels in T2DM mice. Moreover, elevated glucose levels were suppressed and the AUC during the OGTT was decreased, suggesting improved glucose tolerance.

The liver is one of the main organs responsible for regulating blood glucose concentration *via* glycogen metabolism (23). In this study, decreased SOD and CAT levels were seen in the livers of diabetic mice, whereas the probiotic Hao9 significantly reversed this alteration. This finding is consistent with the results of Wu et al. (24). DHE staining and Masson’s trichrome staining also suggested that Hao9 could reduce ROS accumulation in the liver and improve hepatic fibrosis. Glycogen homeostasis is impaired in T2DM mice due to an imbalance between glycogenesis and glycogenolysis (25). Increased endogenous glucose production *via* gluconeogenesis is thought to be the main cause of elevated fasting glucose levels in patients with T2DM (26–28). Hepatic PEPCK and G6Pase are closely associated with hepatic glycogen metabolism disorders in diabetes. G6Pase catalyzes the hydrolysis of glucose-6-phosphate to phosphate and free glucose, which is a critical step in gluconeogenesis and glycogenolysis. Therefore, G6Pase plays a very important role in the regulation of blood glucose levels. PEPCK is a key rate-limiting enzyme in gluconeogenesis (29, 30). Our results suggest that Hao9 can regulate glucose metabolism by increasing the hepatic glycogen content and inhibiting hepatic gluconeogenesis in diabetic mice *via* regulation of these key genes. This finding is consistent with previous studies (24, 31).

Imbalances in the gut microbiome are known to accompany T2DM (2–5). In this study, the gut microbial structure was significantly altered in diabetic mice, and this change was maintained over the course of the experiment. However, as shown by PCoA, the gut microbiota was significantly altered by the Hao9 intervention. As expected, we observed enrichment of *Lacticaseibacillus* in the Hao9 group throughout the experiment (Figures 4, 5), suggesting successful colonization of the gut by Hao9. In addition, the Mantel test revealed a significant correlation of the CTL gut microbiome at weeks 5 and 12, while no significant correlation was observed in the T2DM or Hao9 groups. However, a larger *r* value and a smaller *p*-value was observed for the Hao9 group than for the T2DM group (Figures 7B, C). Therefore, PCoA and Mantel suggested that Hao9 can improve the microbiome composition.

**FIGURE 8:**
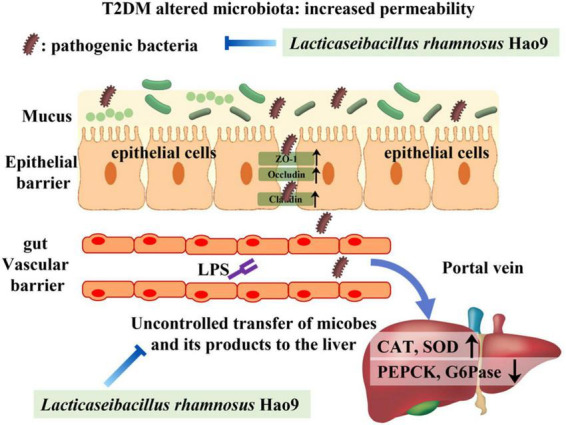
Presumably Hao9 improves the pattern of type 2 diabetes mellitus (T2DM) by modulating gut microbiota.

Dysbiosis of the gut microbiome is thought to play a role in the pathogenesis of T2DM (32, 33). Gut-derived products are transported to the liver *via* the portal vein, with emerging evidence often addressing the relationship between the gut microbiome and the liver (34, 35). LPS is a major component of the cell wall of Gram-negative bacteria and can promote the production of pro-inflammatory cytokines and intestinal barrier damage (36, 37). Intestinal tight junction protein expression is reduced in inflammatory conditions, compromising the integrity of the intestinal barrier (37). Our results indicate that T2DM-induced inflammation and decreases in tight junction protein synthesis were reversed by Hao9 supplementation. Histological analysis of the colon also indicated that Hao9 alleviated colonic damage in T2DM mice. Studies have shown that impaired intestinal barrier function directly promotes T2DM development (6). In addition, systemic inflammation is thought to be an important trigger of insulin resistance (38). Our results suggest that Hao9 acts as an anti-inflammatory probiotic by decreasing serum levels of LPS and pro-inflammatory cytokines (TNFα, IL1β, and IL6), as seen in previous studies (10, 11, 24). Thus, our results suggest that Hao9 may play a role in improving T2DM by remodeling the gut microbiome in diabetic mice. These beneficial effects may be associated with reduced inflammation and improved insulin resistance. Based on these findings, we hypothesize that Hao9 acts as a hypoglycemic agent by improving the composition of the gut microbiome, repairing the intestinal barrier, and relieving inflammation (Figure 8).

In conclusion, this study found that Hao9 supplementation can increase the hepatic antioxidant capacity, alleviate inflammation, and repair intestinal barrier injury in diabetic mice. These effects are associated with improvements in T2DM symptoms. We hypothesize that modulation of the gut microbiome contributes to these effects. Thus, Hao9 supplementation may be a promising strategy for the treatment of T2DM.

## Data availability statement

The datasets for gut microbiota presented in this study can be found in online repositories. The names of the repository and accession number(s) can be found below: https://www.ncbi.nlm.nih.gov/, PRJNA891923.

## Ethics statement

This animal study was reviewed and approved by all procedures involving mice conformed to the guidelines provided by Shanghai Laboratory Animal Center for Animal Care and Animal Experimentation under license number: 2022032002.

## Author contributions

MH and ZG: conception and design of the study and analysis and interpretation of data. WL and YD: collection of samples. MH and YD: laboratory test. MH and CB: drafting of the manuscript. ZG: administrative support and study supervision. All authors read, revised, and approved the final draft.

## Footnotes

1
http://www.drive5.com/usearch/

2
https://www.drive5.com/usearch/manual/sintax_downloads.html

## Supplementary material

Supplementary Figure 1 Periodic acid-Schiff (PAS) straining of colon tissue sections (scale 100 μm).
