# Supplementary figures and images for: Lacticaseibacillus rhamnosus Hao9 exerts antidiabetic effects by regulating gut microbiome, glucagon metabolism, and insulin levels in type 2 diabetic mice
Source: Front Nutr. 2023 Jan 5;9:1081778. doi: 10.3389/fnut.2022.1081778 (PMC9849894; doi:10.3389/fnut.2022.1081778)

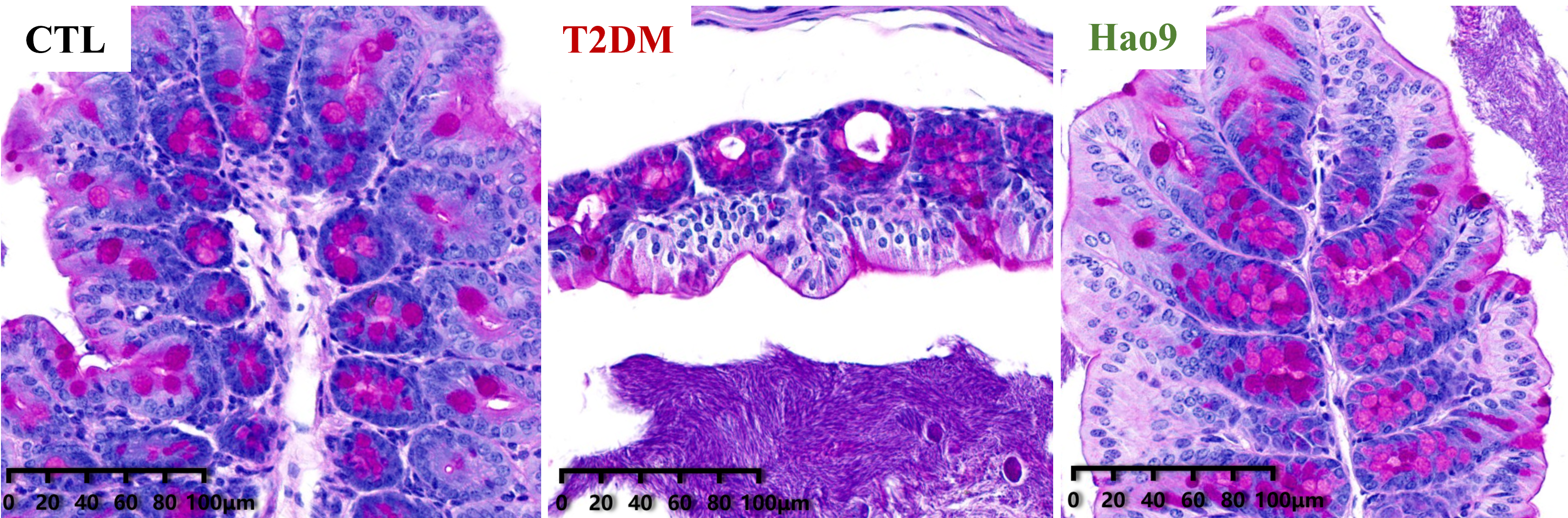

Supplement: Supplementary Figure 1 — Periodic acid-Schiff (PAS) straining of colon tissue sections (scale 100 μm). [file Image_1.TIF]
